# Intrathoracic lipoma in the horizontal fissure of the right lung: a case report

**DOI:** 10.1093/jscr/rjab385

**Published:** 2021-09-09

**Authors:** Eiji Nakajima, Hidenobu Takahashi, Naohiro Kajiwara, Kinya Furukawa, Taichiro Ishizumi, Hiroaki Kataba, Mitsuru Okubo, Hiroshi Hirano, Norihiko Ikeda

**Affiliations:** Department of Thoracic Surgery, Toda Chuo General Hospital, Saitama, Japan; Department of General Thoracic Surgery, Tokyo Medical University Hachioji Medical Center, Tokyo, Japan; Department of General Thoracic Surgery, Tokyo Medical University Hachioji Medical Center, Tokyo, Japan; Department of Thoracic Surgery, Tokyo Medical University Ibaraki Medical Center, Ibaraki, Japan; Department of Thoracic Surgery, Toda Chuo General Hospital, Saitama, Japan; Department of Thoracic Surgery, Toda Chuo General Hospital, Saitama, Japan; Department of Radiology, Tokyo Medical University Hachioji Medical Center, Tokyo, Japan; Department of Diagnostic Pathology, Tokyo Medical University Hachioji Medical Center, Tokyo, Japan; Department of Thoracic Surgery, Tokyo Medical University, Tokyo, Japan

## Abstract

Lipomas are benign tumors that originate from mesenchymal tissue, such as subcutaneous tissue. Intrathoracic lipomas are rare, and they can occur in the chest wall, mediastinum and bronchi. In the present case, the patient had an intrathoracic lipoma that was located in the horizontal fissure of the right lung. Retrospective review of chest radiographs taken at a previous health checkup confirmed that the tumor was growing. The patient had no symptoms, and computed tomography and magnetic resonance imaging suggested that the tumor was a hamartoma. The tumor was resected by video-assisted thoracic surgery, and was diagnosed by pathological analysis as an intrathoracic lipoma consisting of no atypical fats.

## INTRODUCTION

Lipomas are benign tumors comprised of fat tissue. They usually develop subcutaneously, and intrathoracic lipomas are rare [1]. Large intrathoracic lipomas have been reported to cause various symptoms, namely, shortness of breath, chest pain, worsening dyspnea and persistent palpitations [2–5]. In the present case, an intrathoracic lipoma was detected on chest radiography, although the patient did not have any symptoms. Retrospective review of chest radiographs taken at a previous health checkup confirmed that the tumor was growing. The tumor was resected by video-assisted thoracic surgery (VATS), and was confirmed to be an intrathoracic lipoma by pathological analysis.

## CASE REPORT

**Figure 1:**
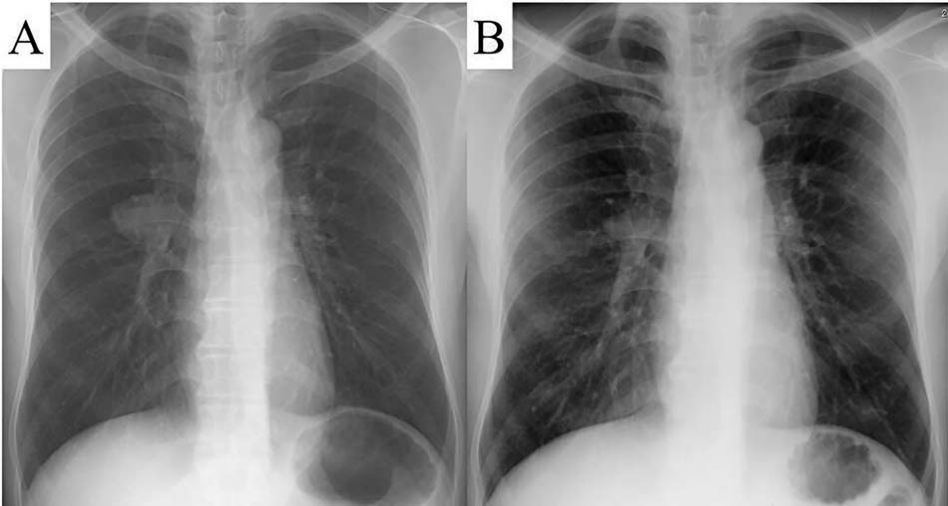
A round mass was detected in the hilum of the right lung by chest radiography (**A**). A chest radiograph taken one and a half years previously displayed a normal view (**B**).

**Figure 2:**
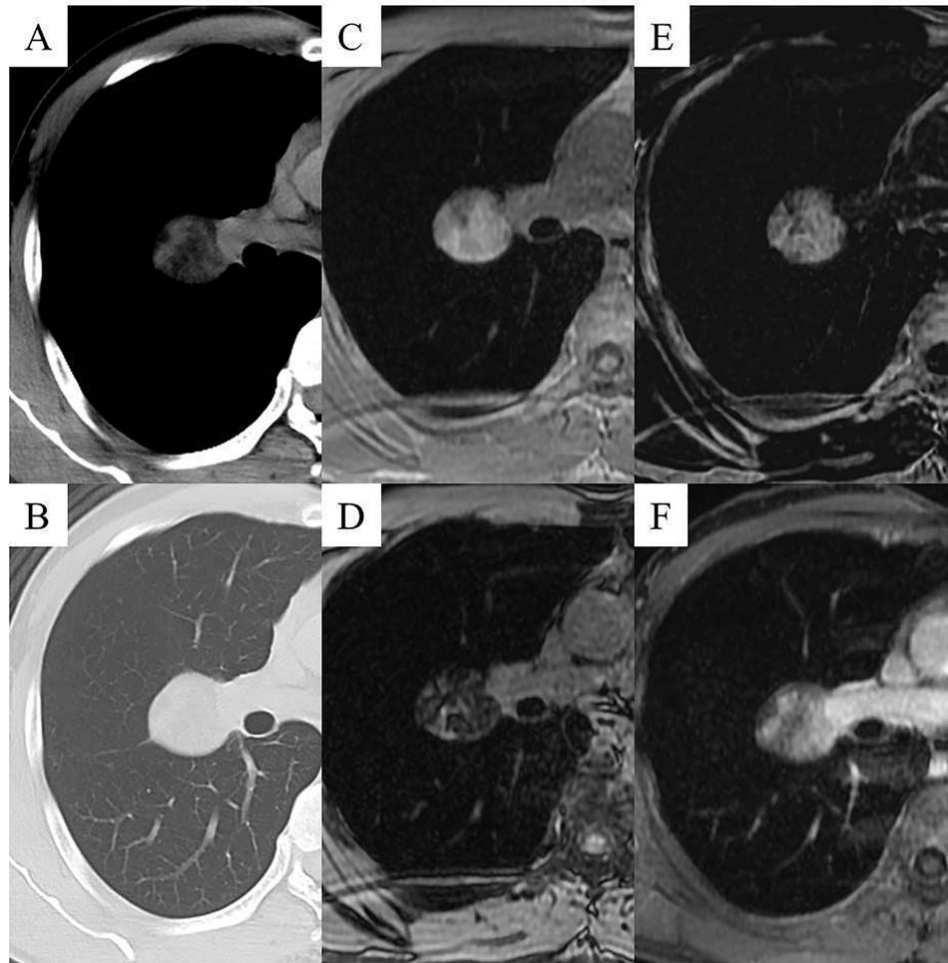
Chest CT images displaying the mass lesion; the low-attenuated mass was displayed in the mediastinal window (**A**), and the diameter of the mass was 3.5 cm and the mass was located close to the fissure of the right lung in the pulmonary window (**B**). Chest MRI displaying the mass lesion; the mass lesion was displayed as a high signal on in-phase T1WI (**C**), the mass was displayed as a low signal on out-of-phase T1WI (**D**), the mass was displayed as a low signal on T2WI (**E**), and the mass lesion was not clearly enhanced by fat-saturated contrast-enhanced T1WI with extracellular gadolinium contrast material (gadoterate meglumine; Magnescope®; Guerbet) (**F**).

The patient was a 48-year-old man, in whom a round mass lesion was detected in the hilum of the right lung by chest radiography performed at an annual health checkup (Fig. 1A). The image of this mass lesion had been interpreted as a normal view on chest radiography performed at his annual health checkup one and a half years previously (Fig. 1B). The mass detected by radiography was also detected by computed tomography (CT), as a 3.5 cm low-attenuated mass with a circumscribed margin in a fissure of the right lung (Fig. 2A, B). On magnetic resonance imaging (MRI), this mass lesion was displayed as a high, low and low signal mass on the in-phase of T1-weighted imaging (WI), out-of-phase of T1WI and T2WI, respectively (Fig. 2C–E). The mass lesion was not clearly enhanced by fat-saturated contrast-enhanced T1WI with extracellular gadolinium contrast material (gadoterate meglumine; Magnescope®; Guerbet, Villepinte, France) (Fig. 2F). The radiological observations were highly suggestive of a hamartoma.

**Figure 3:**
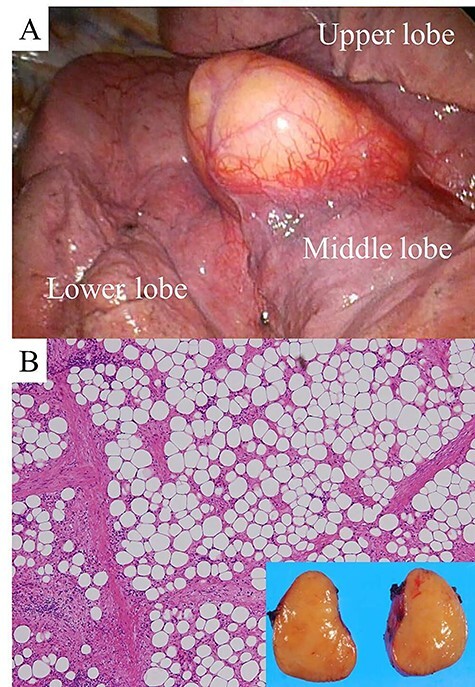
View during VATS (**A**). Hematoxylin and eosin staining of a section of the resected tumor (high magnification). The inset is a macroscopic photograph of the cut surface of the tumor (**B**).

The growing tumor was resected by VATS under general anesthesia with differential pulmonary ventilation. The tumor was located under the visceral pleura on the horizontal fissure of the right lung (Fig. 3A). The size of the capsulated yellow tumor was 3.5 × 3.5 × 2.0 cm. The tumor was microscopically composed of mature adipose tissue separated by fibrous tissue of various widths. The adipocytes were of various sizes, and there was no significant nuclear atypia. Areas of lymphocyte infiltration were found to be separated by connective tissue (Fig. 3B). These findings altogether led to a diagnosis of lipoma.

## DISCUSSION

Lipomas, which originate from adipocytes, are common benign tumors. These tumors are usually located in mesenchymal tissue, such as subcutaneous tissue, and are easily removed for treatment and diagnosis. It is rare for lipomas to grow in the thoracic cavity, and in such cases, diagnosis requires surgical resection of the tumor under general anesthesia. In a previous report, the locations of intrathoracic lipomas were found to be the chest wall, mediastinum and bronchi [1]. In previous cases, massive intrathoracic lipomas causing symptoms were resected by thoracotomy [2–5]. The present patient was a very rare case, in which the tumor was located under the visceral pleura in the horizontal fissure of the right lung. The growing intrathoracic lipoma detected on radiography of the annual health checkup was not causing any symptoms, and was treated by VATS.

## CONCLUSION

Chest radiography performed at an annual health checkup was useful for the detection of a benign growing tumor, which was an intrathoracic lipoma in a rare location.

## References

[1] Sakurai H, Kaji M, Yamazaki K, Suemasu K. Intrathoracic lipomas: their clinicopathological behaviors are not as straightforward as expected. Ann Thorac Surg 2008;86:261–5. 18573434 10.1016/j.athoracsur.2008.03.052

[2] Rahman SMT, Rahim A, Kibria AA. Unusual cause of large intrathoracic mass in a young male of Bangladesh: A case report of giant intrathoracic lipoma & literature review. Int J Surg Case Rep 2020;76:73–6. 33011659 10.1016/j.ijscr.2020.09.164 PMC7530211

[3] Botianu PV, Cerghizan AM, Botianu AM. Giant right intrathoracic myxoid fusocellular lipoma. Case Rep Pulmonol 2015;2015:302189. 26509096 10.1155/2015/302189 PMC4609818

[4] Chen M, Yang J, Zhu L, Zhao H. Intrathoracic giant pleural lipoma: case report and review of the literature. J Cardiothorac Surg 2013;8:196. 24120207 10.1186/1749-8090-8-196 PMC3973812

[5] Asteriou C, Lazopoulos A, Giannoulis N, Kalafatis I, Barbetakis N. Brugada-like ECG pattern due to giant mediastinal lipoma. Hippokratia 2013;17:368–9. 25031519 PMC4097421

